# Electroconvulsive therapy (ECT) and schizophrenia

**DOI:** 10.1192/bjo.2021.872

**Published:** 2021-06-18

**Authors:** Rhys Masin, George Kirov

**Affiliations:** 1 Cardiff University School of Medicine; 2 ECT Department, Cardiff & Vale University Health Board, Cardiff University School of Medicine

## Abstract

**Aims:**

An evaluation of the benefits of ECT in patients with schizophrenia who received ECT in Cardiff & Vale UHB, in order to:

- Investigate the effectiveness of ECT as a treatment for schizophrenia
- Inform future clinical practice
- Identify clear outcome measures for use in future research

**Background:**

Schizophrenia is a severe and debilitating mental illness, for which pharmacological treatment is often ineffective. ECT is seldom used for schizophrenia, despite encouraging evidence for its efficacy. Current guidance in the UK is inconsistent, as RCPsych contemplates the use of ECT in schizophrenia in certain cases, while NICE does not. This confusion warrants the need for further evaluation of ECT as a treatment for schizophrenia.

**Method:**

Eight suitable patients were identified, and a retrospective chart review was conducted in relation to the following outcomes:

- What was the main indication for ECT, and was the issue resolved
- Change in clinical rating scales
- Concordance with medication before and after treatment
- Length of hospital stay before and after treatment, over one year
- Mental Health Act status after treatment for those treated on section
- Was the level of observation reduced following treatment

**Result:**

Initial indication for treatment was completely resolved in seven out of eight cases. All patients improved in overall symptomatic score (mean improvement = 59.5%). Five patients (62.5%) improved above the threshold of clinically significant response. At the commencement of treatment, three (37.5%) patients were refusing all medication, three (37.5%) had poor concordance and two (25%) were fully concordant. At treatment endpoint, all were fully concordant. Average length of hospital stay remained unchanged: 30 weeks during the year before ECT, and 33 weeks during the year after ECT. Of six patients treated under Section 3, four (66.7%) had their section lifted within six months. Observation level was reduced in all cases that had been placed under continuous observation.

**Conclusion:**

ECT improved all outcomes except admission duration. These results provide support for the consideration of ECT as a meaningful treatment option for schizophrenia.

